# Durability of Silicone-Based Waterproofing Membranes in Hempcrete Systems Under Environmental Exposure: Role of Leachate Chemistry and Fiber Treatment

**DOI:** 10.3390/polym18111311

**Published:** 2026-05-26

**Authors:** Elnaz Esmizadeh, Amir Sabziparvar, Marzieh Riahinezhad, Peter Collins, Esrat Jahan, Itzel Lopez-Carreon, Donato Tale Ponga

**Affiliations:** 1 Building Durability and Resiliency, Construction Research Center, National Research Council Canada, 1200 Montreal Rd, Ottawa, ON K1A 0R6, Canada; amir.sabziparvar@nrc-cnrc.gc.ca (A.S.); marzieh.riahinezhad@nrc-cnrc.gc.ca (M.R.); peter.collins@nrc-cnrc.gc.ca (P.C.); esrat.jahan@nrc-cnrc.gc.ca (E.J.);; 2 Department of Civil Engineering, University of Ottawa, 161 Louis Pasteur, Ottawa, ON K1N 9K5, Canada; 3 Center of Innovative Technology and Ecodesign (CITÉ), University of Sherbrooke, Sherbrooke, QC J1K 2R1, Canada

**Keywords:** silicone membrane, hempcrete, accelerated aging, alkalinity, durability

## Abstract

This study investigates the durability of silicone-based membranes in contact with hempcrete under combined moisture and temperature exposure. Membrane specimens were aged in contact with non-treated and treated hempcrete under dry and wet conditions at temperatures up to 90 °C. The evolution of chemical, thermal, and microstructural properties was characterized using FTIR, TGA, DSC, optical microscopy, and SEM–EDS analyses. Results show that dry exposure does not induce measurable changes in membrane structure or performance, confirming that temperature alone is not a critical degradation factor. In contrast, wet exposure leads to significant chemical, thermal, and microstructural changes in the membrane, including degradation of the siloxane network, reduced polymer chain mobility, and the formation of calcium-rich mineral deposits at the interface. These results indicate that membrane degradation is governed by a coupled moisture–ion mechanism involving ion transport, mineral deposition, and hydrolysis of the polymer network. Fiber treatment slightly reduces the aggressiveness of the leachate but does not prevent degradation under wet conditions. Overall, moisture availability and leachate chemistry are identified as key factors controlling the durability of silicone membranes in contact with bio-based materials.

## 1. Introduction

The durability of building envelope materials is a critical factor in long-term building performance and service life [1]. As mentioned in CSA S478 (2019), modern buildings are typically designed for service lives of 50–99 years, during which envelope materials are exposed to various environmental stressors such as temperature cycles, moisture gradients, and chemical aging [2,3]. Among envelope protection systems, polymer-based waterproofing membranes are widely used due to their flexibility and chemical and moisture resistance [4]; however, their durability remains a concern, particularly under aggressive chemical and hygrothermal environments. Silicone-based materials have been extensively used in construction since their introduction in the 1950s [5], initially as joint sealants [6,7] and later as surface coatings and waterproofing membranes [8,9,10]. Silicone materials are valued for their elasticity, high UV resistance, and stability under thermal cycling [11]. Fluid-applied silicone membranes extend these advantages to large-area envelope protection systems by forming seamless vapor-permeable barriers suitable for façades and roofing assemblies. Despite these favorable properties, silicone polymers are still susceptible to long-term chemical and physical aging under aggressive environmental conditions [12].

The durability of silicone materials is governed primarily by the stability of the siloxane (Si–O–Si) backbone, which can undergo hydrolytic or chemically induced chain scission when exposed to moisture, temperature fluctuations, and ionic species. In particular, hydrolysis reactions can be accelerated in environments with extreme pH levels, leading to polymer chain degradation, surface hardening, and loss of mechanical flexibility. Previous studies and manufacturer guidelines indicate that silicone sealants and coatings may be particularly vulnerable to hydrolytic degradation when exposed to environments with extreme pH conditions [13]. In strongly alkaline or acidic media, the siloxane network can be attacked by reactive chemical species, leading to depolymerization and reduced mechanical integrity [14]. More recently, Masson et al. [12] demonstrated that exposure of silicone membranes to a highly alkaline solution simulating concrete pore conditions led to progressive changes in surface and bulk morphology, chemical structure, and toughness, confirming the susceptibility of silicone membranes to strongly alkaline aqueous environments. The susceptibility of silicone polymers to chemical degradation depends on service conditions and the adjacent microenvironment, including temperature, moisture, exposure duration, and substrate chemistry. Consequently, understanding substrate–polymer interactions is essential for assessing compatibility and hence predicting the long-term durability of silicone-based waterproofing systems.

Hempcrete has emerged as a promising low-carbon building material for thermal insulation and envelope applications [15,16]. Hempcrete is a bio-composite produced by combining hemp shives with mineral binders, most commonly lime-based systems. The material is characterized by high porosity, low thermal conductivity, and strong hygroscopic moisture buffering capacity [17]. From a sustainability perspective, hempcrete offers significant environmental advantages due to the carbon sequestration potential of hemp cultivation and the carbonation reactions associated with lime hardening [16].

The performance of hempcrete is governed by the combined effects of binder chemistry and the composition of hemp shives. Binder type dictates the dominant reaction mechanisms, pore solution chemistry, and pH environment. Air lime (hydrated lime) hardens primarily through carbonation with atmospheric CO_2_, resulting in high vapor permeability but relatively low early mechanical strength [16]. In contrast, natural hydraulic lime (NHL) and Ordinary Portland cement (OPC) undergo hydraulic reactions in addition to carbonation, leading to higher early strength and improved mechanical performance [18]. These differences in reaction pathways also influence the pore solution chemistry and moisture transport behavior of the composite.

In parallel, the chemical composition of hemp shives, including cellulose, hemicellulose, lignin, and other extractives, can further modify the microenvironment, particularly when influenced by pre-treatment or degradation processes [19]. The interaction between binder and shives therefore defines the physicochemical environment of hempcrete, controlling pore structure, moisture dynamics, and interfacial characteristics. Consequently, these factors directly influence the conditions at the membrane–substrate interface, including local pH, ionic transport, and moisture state, which are critical to the long-term durability of polymeric waterproofing membranes, particularly silicone-based systems.

Despite increasing adoption of hempcrete in sustainable construction, limited understanding exists regarding the compatibility of polymer waterproofing membranes with lime-based bio-composites. Existing studies on silicone durability primarily focus on simplified alkaline environments or concrete systems, while the combined effects of moisture, ion-rich leachates, and bio-based substrates remain poorly understood. In particular, the role of interfacial moisture–ion interactions and fiber treatment in governing membrane degradation has not been systematically investigated. This knowledge gap is especially important because the high porosity, moisture transport capacity, and chemically active binder system of hempcrete may create interfacial conditions that affect the long-term durability of adjacent polymeric membranes. Therefore, membrane–hempcrete compatibility represents an important durability issue for bio-based envelope systems.

In this study, the long-term durability and chemical stability of a commercially available silicone-based fluid-applied membrane were investigated when applied to hempcrete substrates, with a specific focus on the effect of shiv pre-treatment. Hempcrete samples were produced in the lab using a hydrated lime binder and hemp shives, both non-treated and hydrothermally pre-treated, to assess the influence of shiv chemistry on interfacial conditions. Membrane specimens were subjected to accelerated hygrothermal aging under dry and wet conditions across a temperature range of 22 °C to 90 °C for up to 9 months, simulating realistic service scenarios, including moisture condensation, capillary water transport, and thermal cycling. Membrane degradation was characterized using thermal analysis, chemical spectroscopy, and microstructural imaging techniques. The work aims to elucidate the role of shiv modification in governing silicone membrane performance and to support the design of durable, bio-based building envelope systems.

## 2. Experimental

### 2.1. Materials and Methods

The waterproofing membrane used in this study was a commercially available silicone-based coating, DOWSIL™ AllGuard Silicone Elastomeric Coating (The Dow Chemical Company, Midland, MI, USA) [20]. The key material specifications provided by the manufacturer are summarized in Table 1. The product is a single-component, water-based silicone elastomer formulated for above-grade exterior masonry applications. The material cures upon drying to form a continuous, elastomeric, vapor-permeable membrane. In this study, the silicone coating was applied using a roller in five successive layers to achieve a minimum dry film thickness of 1.0 mm. Each layer was allowed to dry for 4–8 h prior to subsequent application to ensure adequate interlayer adhesion, uniform membrane formation and long-term waterproofing performance.

The hempcrete specimens were produced in the laboratory using a hydrated lime binder (Mortaseal) and hemp shives. The Mortaseal binder primarily consists of calcium oxide (CaO) and magnesium oxide (MgO), as examined by X-ray fluorescence (XRF) in the lab. The volumetric mix proportion of hemp shives, binder, and water was 1:0.24:0.35, respectively. This formulation was selected based on preliminary trials and literature-reported hempcrete ranges [21] to provide a workable, cohesive mixture while balancing density, thermal-insulation potential, and mechanical stability for the intended application. Two types of hemp shives were incorporated into the mixtures, non-treated (NT) and treated (T), and these designations are used hereafter to identify the corresponding hempcrete samples. The treated hemp shives were subjected to a hydrothermal treatment, in which they were soaked and stirred in deionized water at 90 °C for 2 h. This treatment was applied to partially remove water-soluble extractives and other shiv-derived constituents that may influence hemp–binder interactions and leachate chemistry. In the present study, it was used to assess whether modifying the chemical contribution of the hemp shives affects the interfacial environment and subsequent membrane degradation.

### 2.2. Aging Protocol

**Figure 1:**
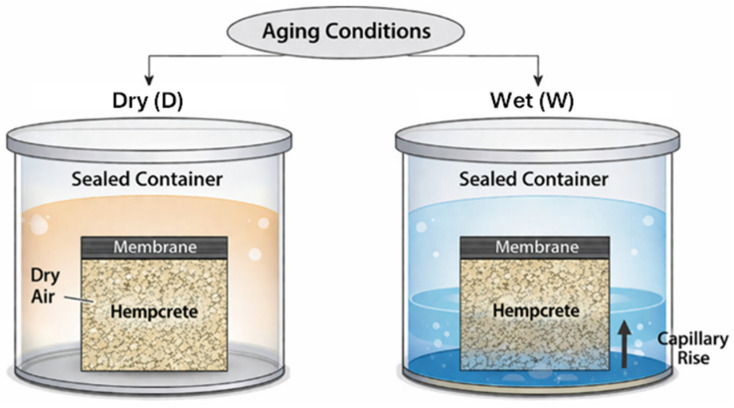
Schematic of the membrane–hempcrete assembly under dry (D) and wet (W) aging conditions at RT, 50 °C, 70 °C, and 90 °C. In the wet condition, water (≈2/3 height) induces capillary rise within the hempcrete, maintaining a moist interface with the membrane. (Image generated using AI (Copilot (https://copilot.microsoft.com/)) and edited by the authors).

To simulate realistic in-service conditions, free-standing membrane samples were positioned in direct contact with hempcrete substrates and exposed to controlled aging environments under both dry (D) and moisture-conditioned wet (W) states. Aging experiments were performed at room temperature (RT) and at elevated temperatures of 50 °C, 70 °C, and 90 °C. Under dry exposure, membrane–hempcrete assemblies were sealed in enclosed containers without added water, allowing evaluation of thermally driven degradation while limiting moisture-related chemical interactions. For wet exposure, the assemblies were placed in sealed containers containing water to approximately two-thirds of the hempcrete height. This water level was selected to supply moisture through capillary rise without fully immersing the membrane, thereby allowing the exposure to be governed by substrate-mediated moisture transport (Figure 1). The wet condition was designed to replicate environmental scenarios such as precipitation ingress, condensation, or localized leakage, enabling assessment of the combined influence of moisture and temperature on degradation mechanisms.

Sample nomenclature reflects hempcrete treatment, exposure condition, and aging temperature. Hempcrete fabricated with non-treated shives is denoted as NT, while those made out of the treated shives are indicated as T. The letters D and W represent dry and wet conditions, respectively. The full sample nomenclature is summarized in Table 2. For example, HWNT-70 refers to a membrane specimen aged in contact with non-treated hempcrete under wet exposure at 70 °C.

Samples were retrieved after 3, 6, and 9 months of exposure for characterization. Prior to testing, all specimens were conditioned at RT for at least 24 h to ensure thermal equilibration. In cases where mold was observed on hempcrete before a scheduled sampling interval, the exposure duration at failure was recorded as the terminal aging time, and further aging was discontinued due to health and safety considerations.

Because material degradation at typical service temperatures proceeds slowly, elevated temperatures (50 °C, 70 °C, and 90 °C) were employed to accelerate aging reactions. Although these temperatures exceed normal field conditions, they facilitate thermally activated degradation processes consistent with Arrhenius behavior, enabling extrapolation of long-term performance trends. Aging processes were conducted using temperature-regulated convection ovens (FED 720, BINDER GmbH, Tuttlingen, Germany) and a constant climate chamber (HPP260, Memmert GmbH + Co. KG, Schwabach, Germany) to maintain stable experimental conditions throughout the aging period.

### 2.3. Characterization

Membrane specimens aged in contact with hempcrete under the various exposure conditions were characterized using a range of analytical techniques to evaluate potential degradation relative to the non-aged reference sample.

#### 2.3.1. pH Monitoring of Wet-Aging Medium

pH measurements were performed on a weekly basis using an PH100 pH meter (Extech Instruments, Nashua, NH, USA) to monitor the alkalinity of the liquid stored in the wet-aging containers. Tracking pH changes over time was important for evaluating their potential influence on membrane degradation mechanisms. The pH of the tap water used to create the wet condition was approximately 9.5 ± 0.3.

#### 2.3.2. Thermal Analyses

Thermogravimetric analysis (TGA) was performed using a Discovery TGA5500 (TA Instruments, New Castle, DE, USA). Approximately 10 mg of each membrane sample was heated in platinum pans from 40 °C to 800 °C at 10 °C/min under an air atmosphere. The analysis was conducted to assess changes in thermal stability and mass loss behavior associated with degradation. Differential scanning calorimetry (DSC) measurements were also carried out using a Discovery DSC2500 (TA Instruments, New Castle, DE, USA) to determine melting temperature (T_m_) and crystallization temperature (T_c_). Approximately 5 mg of each sample was placed in T_zero_ aluminum pans and analyzed under a nitrogen atmosphere. The thermal program consisted of two heating cycles: an initial heating from 40 °C to 100 °C to remove any prior thermal history, followed by cooling to −150 °C and a second heating to 100 °C. All heating and cooling steps were conducted at a rate of 10 °C/min.

#### 2.3.3. Fourier-Transform Infrared Spectroscopy (FTIR)

Fourier-Transform Infrared Spectroscopy (FTIR) was conducted to evaluate chemical degradation by observing changes in the characteristic absorption bands of functional groups. The spectra were obtained using a Nicolet iS50R FTIR spectrometer (Thermo Fisher Scientific, Waltham, MA, USA) equipped with an attenuated total reflectance (ATR) accessory containing a single-reflection diamond crystal positioned at a 45° angle to enhance spectral quality. Spectral data were collected across a wavenumber range of 4000 to 400 cm^−1^, with 32 scans averaged at a spectral resolution of 4 cm^−1^. To ensure data reliability, at least three replicates were measured for each aging condition. Prior to analysis, all spectra underwent ATR and baseline corrections and were subsequently normalized using the 2960 cm^−1^ peak to allow for consistent comparison between samples.

#### 2.3.4. Visual and Microscopic Observations

Surface morphology of the membrane was examined using a Stemi SV11 optical microscope (Carl Zeiss Microscopy GmbH, Jena, Germany) coupled with a Clemex 1.3C digital camera at 20× magnification to capture microscale surface features. Higher-resolution morphological analysis was also conducted using a S-4800 field-emission scanning electron microscope (FE-SEM) (Hitachi High-Tech Corporation, Tokyo, Japan) operating in secondary electron mode at an accelerating voltage of 1 kV. Before imaging, samples were mounted using carbon adhesive paint to improve electrical conductivity and imaging quality. In addition, the elemental composition of the membrane surface was evaluated using an Ultim Max 170 energy-dispersive X-ray spectroscopy (EDS) detector (Oxford Instruments NanoAnalysis, High Wycombe, UK) performed at an accelerating voltage of 15 kV.

## 3. Results and Discussion

### 3.1. pH Measurements

**Figure 2:**
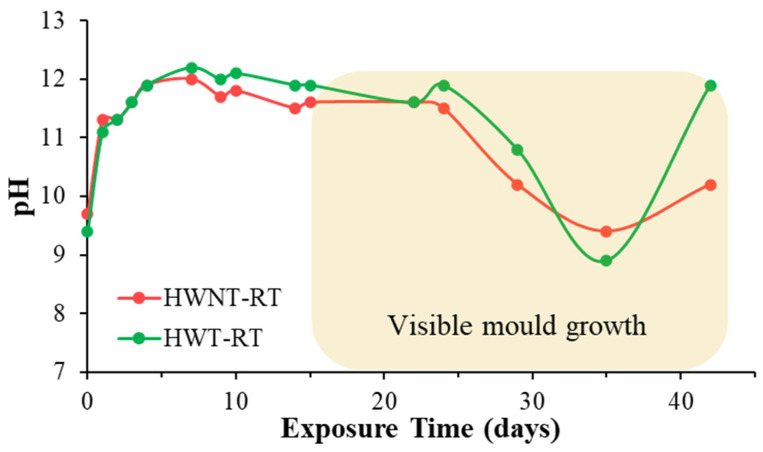
pH changes in the suspensions leached out of the wet samples at RT. Significant visible mold started to appear on the hempcrete as early as 14 days.

The evolution of pH in the aqueous suspensions in contact with hempcrete specimens stored under wet conditions at RT is presented in Figure 2. The suspension was initially tap water; however, its pH changed over time due to the progressive release of leachates from the hempcrete. The results indicate that the pH of the surrounding environment increased rapidly to approximately 11 within the first two days due to leaching from the hempcrete and remained relatively stable thereafter, indicating a consistently strong alkaline system (pH 11–12). Due to rapid mold growth on the hempcrete specimens at RT, with visible fungal development within ~14 days, long-term exposure and subsequent characterization were discontinued due to safety concerns. Nevertheless, the high pH indicates that the alkalinity of the system is primarily controlled by the dissolution of hydroxide-bearing phases in the binder.

**Figure 3:**
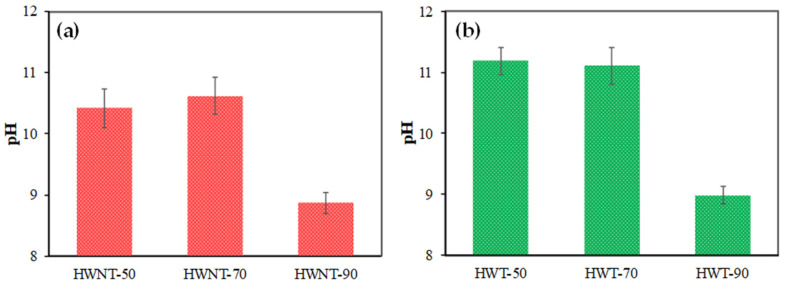
Average pH of leachate suspensions from (**a**) non-treated (HWNT) and (**b**) treated (HWT) hempcrete at elevated temperatures (50, 70, and 90 °C). Values represent weekly averages over a 9-month aging period.

The average pH values of the suspensions at elevated temperatures for both non-treated (HWNT) and treated (HWT) hempcrete are shown in Figure 3, while the corresponding weekly measurements are included in the Supplementary Materials. For these conditions, aging was conducted over an extended period of up to 9 months, and the reported values represent the weekly average of pH measurements collected throughout this period. The results indicate that temperature and shiv treatment have a pronounced effect on leachate chemistry, which in turn influences pH. At 50 °C, the pH of HWNT samples was 10.4 ± 0.3, while HWT samples exhibited a higher pH of 11.8 ± 0.4. A similar trend, with treated samples showing higher alkalinity, was observed at 70 °C, where pH values were 10.6 ± 0.3 for HWNT and 11.2 ± 0.2 for HWT. However, increasing the temperature to 90 °C resulted in a substantial decrease in pH for both materials, reaching 8.9 ± 0.2 for HWNT and 9.0 ± 0.1 for HWT.

**Figure 4:**
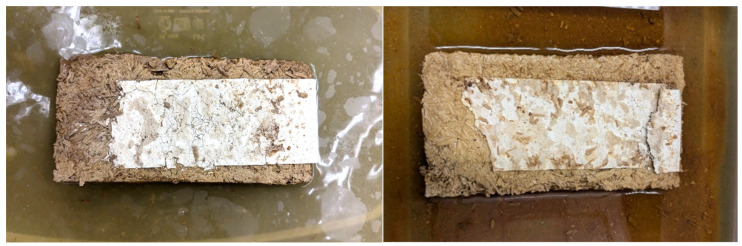
Images of membrane being aged in contact with HWNT (**left**) and HWT (**right**) at 90 °C.

The chemistry of the suspension is influenced by two competing mechanisms: (i) continued leaching of alkaline species from the hydrated binder, and (ii) increased release of acidic organic compounds from hemp shives. These include hemicellulose- and lignin-derived compounds, which contribute to a reduction in pH. This was evidenced by the yellowish color of the suspension leached out of the hempcrete (Figure 4). The combined effect of these opposing processes results in a moderate decrease in alkalinity for samples aged under 50 °C and 70 °C compared to RT conditions. Treated hempcrete samples consistently exhibited slightly higher pH values than non-treated samples across these temperatures. This behavior is attributed to the removal of a portion of acidic extractives during pre-treatment, thereby reducing their contribution to the leachate chemistry and allowing the alkaline binder phases to dominate.

At 90 °C, the influence of acidic extractives becomes dominant for both treated and non-treated samples, leading to a marked reduction in pH. This behavior may be attributed to accelerated thermal degradation of hemp shiv constituents, particularly hemicellulose, which can release organic acids such as acetic acid, as well as other low-molecular-weight and phenolic compounds [22]. As a result, the leachate chemistry shifts from binder-dominated alkalinity toward a stronger contribution from thermally generated organic species, causing the pH to drop to mildly alkaline conditions (≈8–9). The similar pH range observed for treated and non-treated samples at 90 °C suggests that, at this temperature, newly generated degradation products may outweigh the initial reduction in soluble extractives achieved by hydrothermal treatment.

### 3.2. Thermogravimetric Analysis

**Figure 5:**
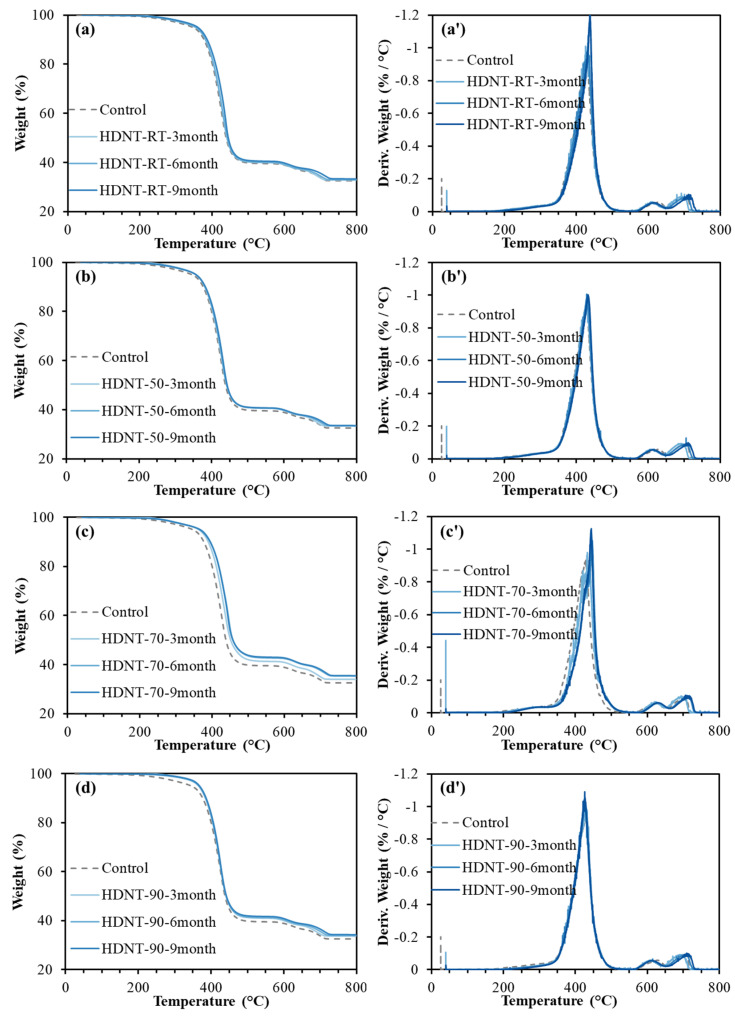
Thermal degradation of the membrane aged in contact with HDNT at different temperatures: (**a**) TGA at RT; (**a’**) DTG at RT; (**b**) TGA at 50 °C; (**b’**) DTG at 50 °C; (**c**) TGA at 70 °C; (**c’**) DTG at 70 °C; (**d**) TGA at 90 °C; and (**d’**) DTG at 90 °C.

**Figure 6:**
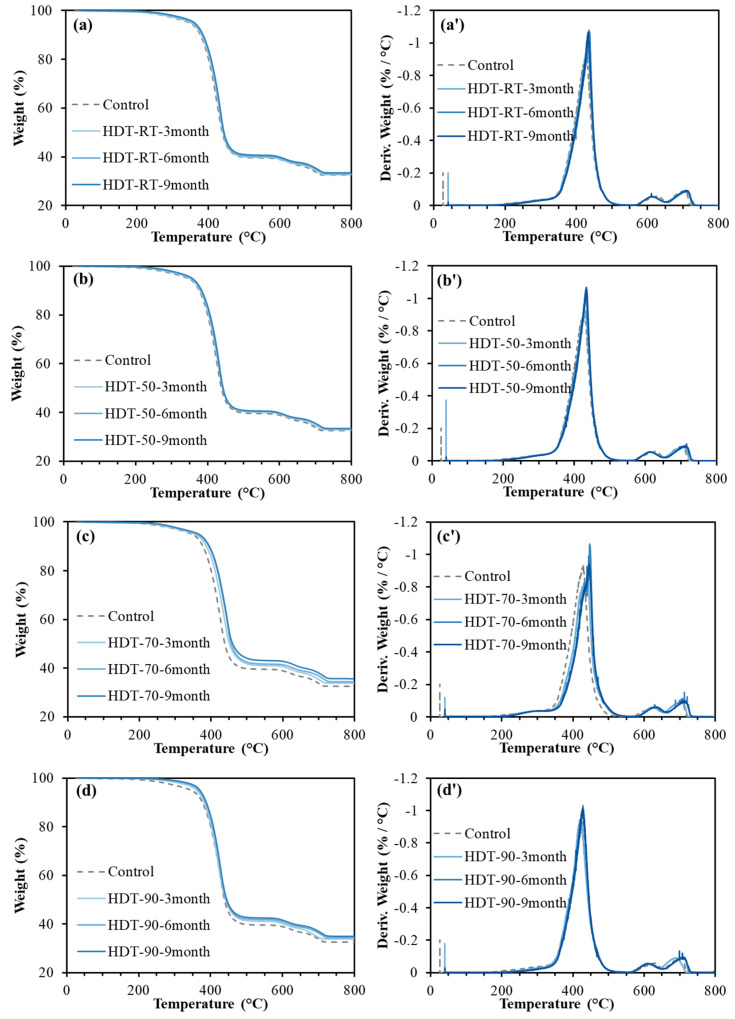
Thermal degradation of the membrane aged in contact with HDT at different temperatures: (**a**) TGA at RT; (**a’**) DTG at RT; (**b**) TGA at 50 °C; (**b’**) DTG at 50 °C; (**c**) TGA at 70 °C; (**c’**) DTG at 70 °C; (**d**) TGA at 90 °C; and (**d’**) DTG at 90 °C.

TGA and derivative thermogravimetric (DTG) analyses of membranes exposed to hempcrete under dry conditions are presented for both non-treated and treated systems in Figure 5 and Figure 6, respectively. The control (non-aged) membrane shows two characteristic decomposition stages typical of silicone-based materials. The primary mass-loss event, with DTG peak at approximately 428 °C, is attributed to degradation of the PDMS network through Si–O bond cleavage and the evolution of volatile cyclic siloxane species [23]. A secondary multi-step decomposition occurs at higher temperatures, with DTG peaks in a range of 625 to 702 °C, corresponding to the thermal dissociation of calcium carbonate (CaCO_3_) fillers. The remaining residue at elevated temperatures is associated with thermally stable inorganic components, such as titanium dioxide (TiO_2_), which do not undergo significant decomposition within the tested range. Membranes aged in contact with hempcrete under dry conditions (HDNT and HDT) exhibit negligible changes in their TGA/DTG profiles, even after prolonged exposure (up to 9 months). This indicates that thermal exposure alone, up to 90 °C, does not induce any significant chemical or structural alteration of the membrane when in contact with the hempcrete surface. This also suggests that degradation mechanisms such as depolymerization or chain scission are not activated under dry conditions. This behavior can be attributed to the high bond energy of the Si–O backbone in PDMS, which confers intrinsic thermal stability, and to the absence of moisture, which suppresses hydrolytic pathways and limits ionic mobility at the membrane–substrate interface [24].

**Figure 7:**
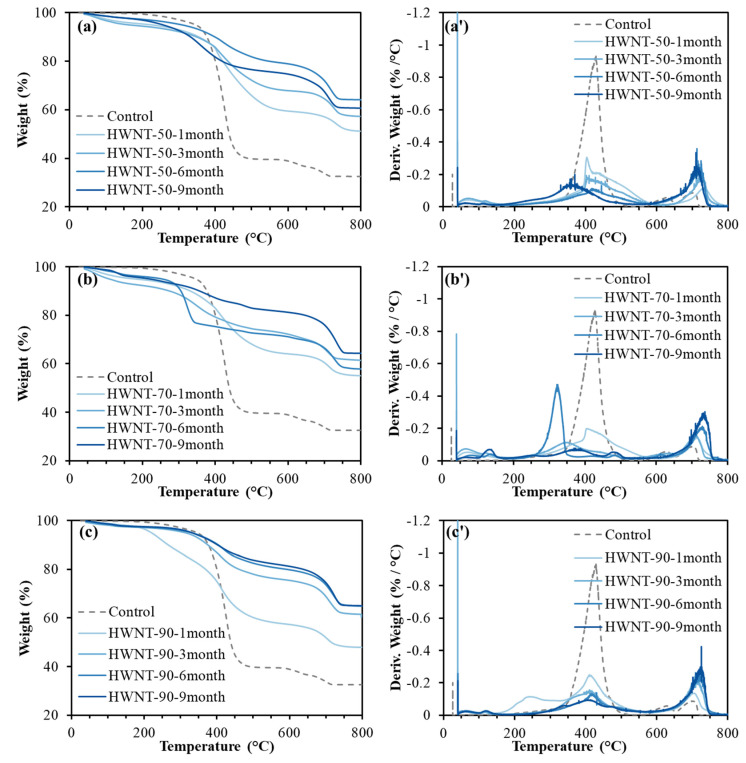
Thermal degradation of the membrane aged in contact with HWNT at different temperatures: (**a**) TGA at 50 °C; (**a’**) DTG at 50 °C; (**b**) TGA at 70 °C; (**b’**) DTG at 70 °C; (**c**) TGA at 90 °C; and (**c’**) DTG at 90 °C.

**Figure 8:**
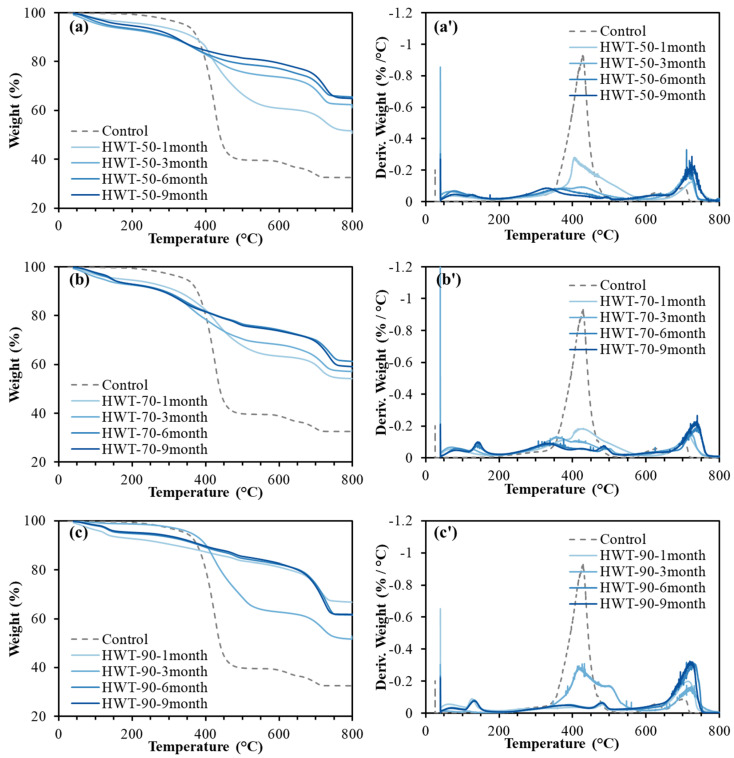
Thermal degradation of the membrane aged in contact with HWT at different temperatures: (**a**) TGA at 50 °C; (**a’**) DTG at 50 °C; (**b**) TGA at 70 °C; (**b’**) DTG at 70 °C; (**c**) TGA at 90 °C; and (**c’**) DTG at 90 °C.

TGA/DTG analyses of membranes exposed to hempcrete under wet conditions (HWNT and HWT), presented in Figure 7 and Figure 8, reveal severe degradation compared to the control and dry-aged samples. Irrespective of aging temperature (50, 70, or 90 °C) or shives pre-treatment condition of the hempcrete, the characteristic DTG peak associated with the PDMS backbone (~430 °C) is significantly reduced or nearly absent. This observation indicates extensive degradation of the siloxane network, suggesting substantial disruption of the polymer structure during exposure to moisture-conditioned hempcrete. At the same time, the intensity of the high-temperature DTG peak attributed to carbonate decomposition (≈700–730 °C) increased markedly, accompanied by a higher residual mass at the end of the test, which suggests the deposition of thermally stable inorganic phases on the membrane surface.

These observations indicate that the degradation of the membrane is governed by chemically aggressive interactions with the hempcrete leachate under wet conditions. While the measured pH of the leachates ranged from moderately to highly alkaline (pH ≈ 9–11), the extent of degradation does not correlate directly with alkalinity alone. Significant degradation was observed even in samples exposed to lower pH conditions (≈9), suggesting that hydroxyl concentration is not the sole controlling factor. Instead, the results suggest that degradation is driven by a coupled moisture–ion mechanism, in which dissolved species such as Ca^2+^ and Mg^2+^, abundant in the hydrated binder, may contribute to interfacial reactions.

In aqueous environments, hydroxyl ions (OH^−^) initiate nucleophilic attack on the siloxane (Si–O–Si) backbone, leading to bond cleavage and the formation of silanol groups (Si–OH) [25]. This hydrolysis process is reported to be accelerated in the presence of divalent cations such as Ca^2+^ and Mg^2+^, although their specific contribution cannot be isolated in the present hempcrete-contact system. These cations act as Lewis acids, coordinating with oxygen atoms in the siloxane network and increasing the polarization of the Si–O bond, rendering the silicon center more susceptible to nucleophilic attack. This interaction lowers the activation energy required for bond cleavage and promotes depolymerization of the PDMS network into lower-molecular-weight species. Consequently, the combined effects of moisture, hydroxyl ions, and divalent cations result in rapid destabilization and breakdown of the polymer backbone, even under moderate alkaline conditions. This interpretation is consistent with previous studies, which have shown that PDMS degradation in alkaline environments is not governed solely by pH, but is strongly influenced by the nature of the cations present. In particular, Ca^2+^ has been reported to promote significantly higher degradation rates compared to Na^+^, even at similar pH levels, indicating a catalytic effect of divalent cations on siloxane bond cleavage and depolymerization processes [25].

In addition to polymer degradation, the increased intensity of carbonate-related DTG peaks and higher residual mass indicate secondary mineral deposition on the membrane surface. In calcium-rich leachates, dissolved Ca^2+^ can react with carbonate species (e.g., CO_3_^2−^ formed via dissolution of atmospheric CO_2_) to precipitate calcium carbonate (CaCO_3_). These precipitates accumulate on the membrane surface, contributing to the increased residual mass observed in TGA and the enhanced peaks in the 700–730 °C range. Such mineral deposits may further influence degradation by modifying the local chemical environment and promoting localized reactions at the polymer–hempcrete interface.

Overall, the results demonstrate that membrane degradation under wet exposure conditions is controlled by coupled physicochemical processes, in which moisture-driven hydrolysis and ion-assisted chemical reactions act synergistically rather than by alkalinity alone. Water facilitates the transport and accessibility of reactive species. Dissolved ions, particularly divalent cations, may further modify the interfacial chemistry and contribute to siloxane bond cleavage. This interplay highlights the critical role of leachate composition in controlling reaction kinetics and pathways, particularly the presence of divalent cations, underscoring that the long-term stability of silicone-based membranes in contact with hempcrete is strongly dependent on the combined effects of moisture state and ionic environment. Further work involving controlled solution-based experiments at constant pH with systematically varied Ca^2+^ and Mg^2+^ concentrations is required to quantitatively isolate their individual effects on silicone degradation.

### 3.3. Fourier-Transform Infrared

**Figure 9:**
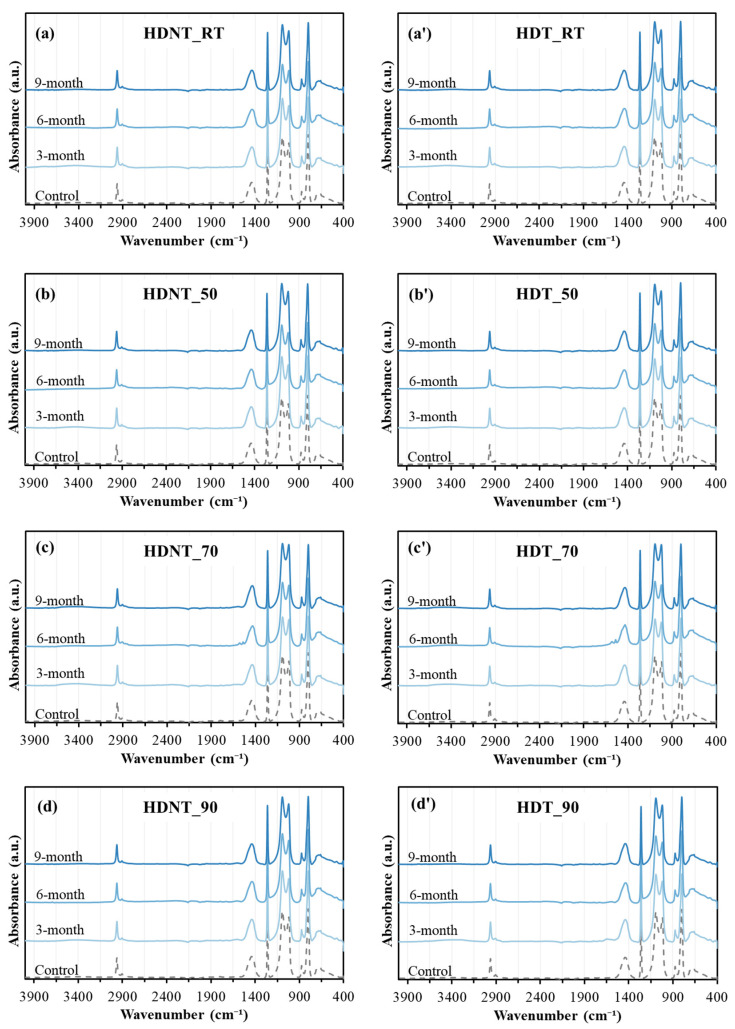
FTIR spectra for the membrane exposed to HDNT (**a**–**d**) and HDT (**a′**–**d′**) at RT, 50 °C, 70 °C, and 90 °C.

Figure 9 shows the FTIR spectra of membranes aged in contact with both non-treated and treated hempcrete (HDNT and HDT) under dry conditions. The spectra for the control membrane exhibit characteristic PDMS absorption bands, including peaks at ~2960 cm^−1^ (C–H stretching of CH_3_ groups), 1260 cm^−1^ (Si–CH_3_ bending), and 800 cm^−1^ (Si–CH_3_ rocking). The strong bands in the 1000–1100 cm^−1^ region correspond to Si–O–Si stretching vibrations, with contributions from Si–O–C linkages [26]. In addition, the presence of the ~1440 cm^−1^ band in the control sample confirms that calcium carbonate is an inherent filler in the membrane formulation.

Consistent with the TGA results, no significant structural changes are observed for membranes aged in contact with either non-treated or treated hempcrete (HDNT and HDT) under dry conditions, as evidenced by the strong overlap between the aged and control spectra. This indicates that, in the absence of moisture, prolonged contact with hempcrete does not induce degradation of the silicone membrane, and its structural integrity, and thus durability, is preserved.

**Figure 10:**
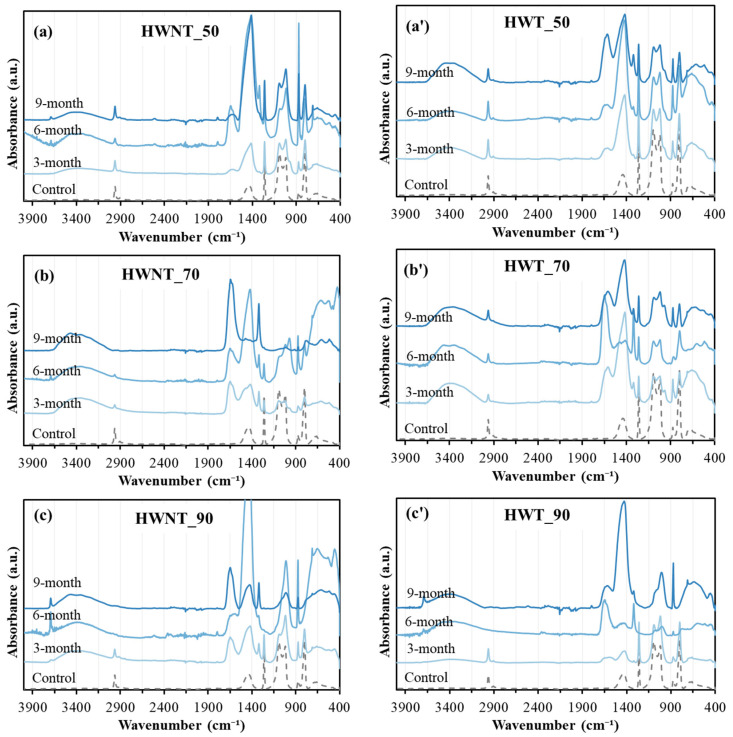
FTIR spectra for the membrane exposed to HWNT (**a**–**c**) and HWT (**a′**–**c′**) at 50 °C, 70 °C, and 90 °C.

In contrast, Figure 10 shows clear chemical changes in membranes exposed to wet hempcrete. Table 3 also summarizes the presence and intensity of the main FTIR bands, while Table 4 provides their chemical assignments. The appearance of O–H (~3450 cm^−1^) and H–O–H (~1640 cm^−1^) bands confirms moisture uptake across all samples under wet exposure. This is accompanied by attenuation or partial disappearance of PDMS-related bands (1260, 1100–1000, and ~790 cm^−1^), indicating degradation of the siloxane network. These changes generally become more pronounced with increasing exposure time and temperature, although some spectral variability is observed due to surface heterogeneity and mineral deposition. The persistent carbonate-related band at ~1440 cm^−1^ further supports the presence of calcium carbonate filler and/or secondary carbonate deposition at the membrane surface. Compared with HWNT samples, HWT samples generally retain PDMS-related bands more consistently, suggesting that hydrothermal treatment slightly reduces leachate aggressiveness, likely through partial removal of soluble extractives. Overall, the FTIR results are consistent with the TGA results, where attenuation of siloxane-related bands corresponds to the reduction or disappearance of the characteristic PDMS decomposition peak (~430 °C). These findings confirm that wet exposure promotes degradation of the silicone membrane, while hemp shiv treatment mitigates but does not prevent this process.

### 3.4. Differential Scanning Calorimetry (DSC)

**Figure 11:**
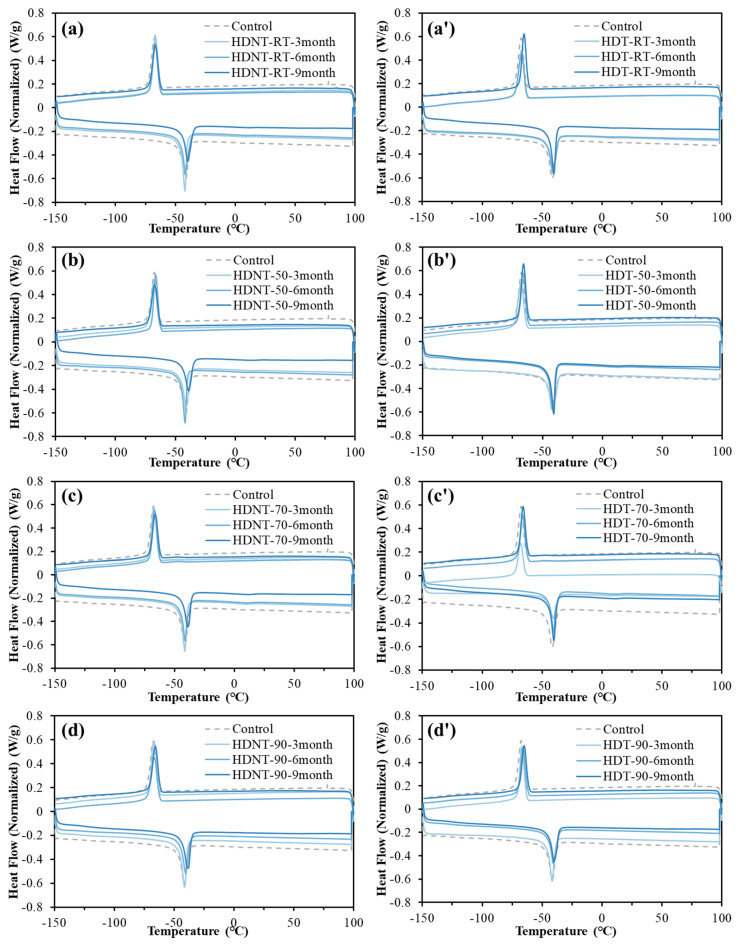
DSC curves, including cooling cycle (or crystallization behavior), and heating cycle (or melting behavior) for HDNT (**a**–**d**) and HDT (**a′**–**d′**) aged at RT, 50 °C, 70 °C, and 90 °C.

**Figure 12:**
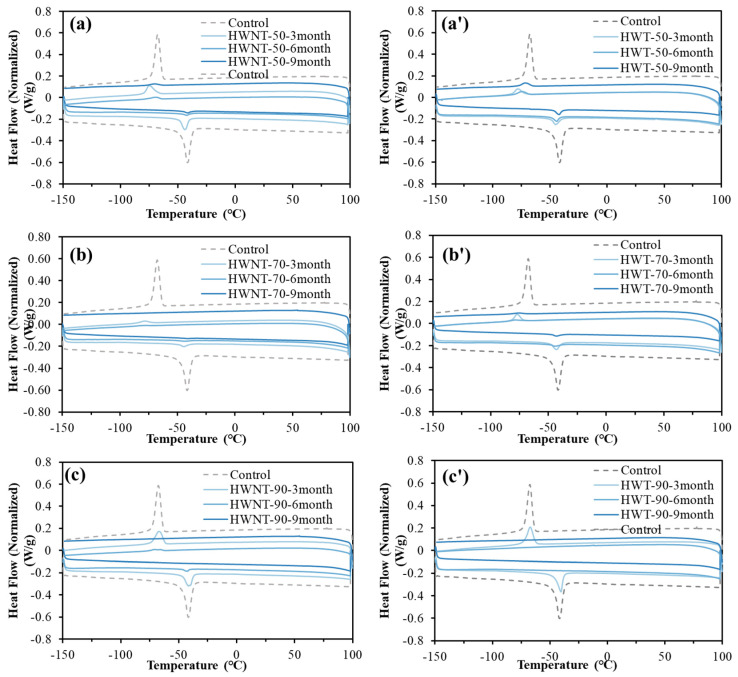
DSC curves, including cooling cycle (or crystallization behavior), and heating cycle (or melting behavior) for HWNT (**a**–**c**) and HWT (**a′**–**c′**) aged at 50 °C, 70 °C, and 90 °C.

DSC results for membranes exposed under dry conditions are presented in Figure 11. However, no measurable differences were observed relative to the control sample, indicating that thermal exposure alone does not alter the structure or thermal behavior of the silicone membrane. In contrast, membranes exposed to hempcrete under wet conditions exhibit clear and progressive changes in their thermal response, as shown in Figure 12. The control sample displays a distinct melting transition (T_m_) at approximately −41 °C and a crystallization transition (T_c_) near −68 °C, characteristic of silicone elastomers [27]. Upon wet exposure, these thermal transitions become progressively less defined with increasing aging duration, exhibiting noticeable reductions at 3 months and significant attenuation and broadening at 6 and 9 months. This effect is further pronounced at elevated temperatures (70–90 °C), indicating that temperature acts as an accelerating factor for degradation processes rather than the primary driving mechanism. The suppression and broadening of these thermal transitions reflect a reduction in polymer chain mobility and integrity. This behavior can be attributed to two concurrent mechanisms. First, chemical degradation of the siloxane backbone through hydrolysis and depolymerization in the presence of moisture and ion-rich leachates, leading to chain scission and molecular weight reduction. Secondly, physical restriction of chain mobility due to the formation and accumulation of inorganic deposits at or near the membrane surface, which impose additional constraints on segmental motion.

Overall, the progressive loss of the characteristic PDMS thermal signature with increasing exposure severity is consistent with FTIR evidence of siloxane bond cleavage and TGA results showing the disappearance of the PDMS decomposition peak. This agreement confirms that membrane deterioration is governed by coupled moisture–chemical interactions, with temperature primarily enhancing reaction kinetics rather than independently inducing degradation.

### 3.5. Optical Micrographs

**Figure 13:**
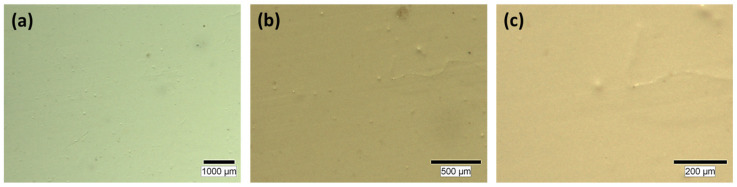
Optical micrographs of the control silicone membrane at (**a**) 5×, (**b**) 10×, and (**c**) 25×.

**Figure 14:**
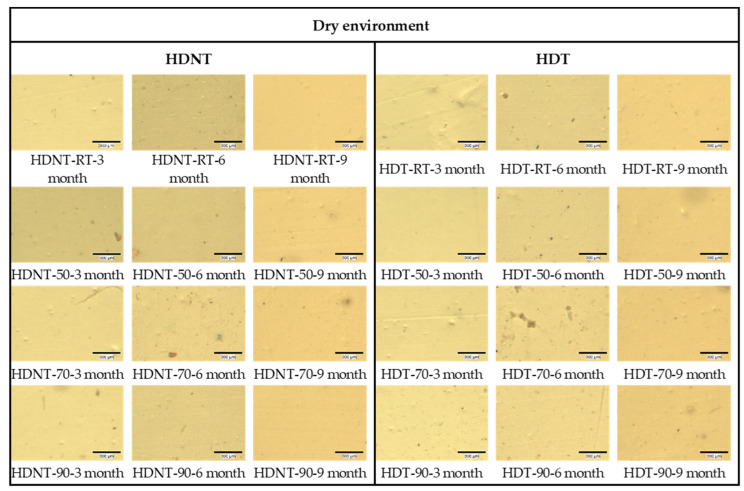
Optical micrographs of aged silicone membranes exposed to HDNT and HDT (25×).

Figure 13 presents the optical micrographs of the non-aged silicone membrane surfaces, showing a relatively uniform surface with minor irregularities such as wrinkles and small blister-like features arising from the coating application process. In comparison, Figure 14 shows membranes aged under dry conditions (HDNT and HDT), which exhibit no significant morphological changes relative to the control, indicating that the surface remains largely unchanged after aging. Any observed color variations across the images are attributed to imaging conditions rather than actual material differences. Similar features are observed in the dry-aged samples, with no systematic changes in morphology or surface integrity. Any slight roughness detected appears random and unrelated to exposure conditions. Overall, the membrane maintains a smooth and continuous surface across all temperatures, indicating that dry contact with hempcrete does not induce detectable surface degradation. This observation is consistent with the absence of chemical and thermal changes identified by FTIR, TGA, and DSC analyses.

**Figure 15:**
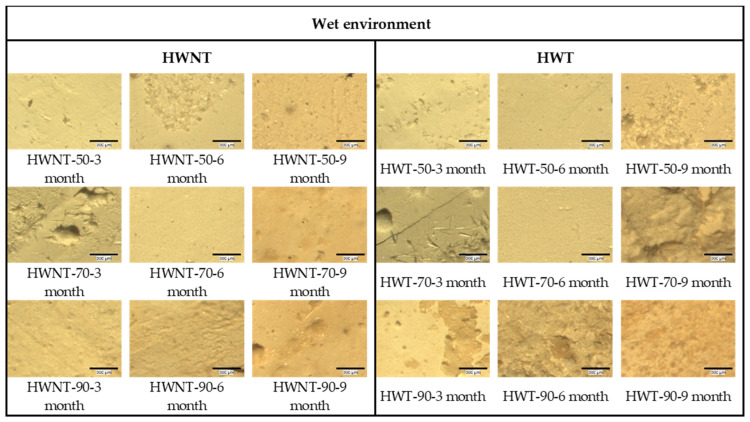
Optical micrographs of silicone membranes exposed to HWNT and HWT (25×).

Optical micrographs of silicone membranes exposed to wet hempcrete, illustrated in Figure 15, reveal clear surface modifications compared to the control and dry-aged samples. Wet-exposed specimens exhibit increased heterogeneity, including the formation of particulate deposits, irregular clusters, and localized surface disruptions. These features become more pronounced with increasing exposure time and temperature, with evidence of micro-scale roughening and the development of crystalline or granular structures distributed across the surface. The morphology suggests both occasional surface deposition and modifications of the underlying polymer structure.

These observations are consistent with the chemical and thermal changes identified by the complementary techniques presented in the previous sections. The presence of particulate and crystalline features aligns with FTIR results showing persistent or enhanced carbonate-related bands and TGA results indicating an increase in inorganic residue, supporting the occurrence of mineral deposition from ion-rich leachates. At the same time, the loss of surface uniformity and emergence of micro-scale defects are indicative of polymer degradation, consistent with the attenuation of PDMS-related FTIR peaks and the suppression of thermal transitions observed in DSC.

### 3.6. Scanning Electron Microscopy Images

**Figure 16:**
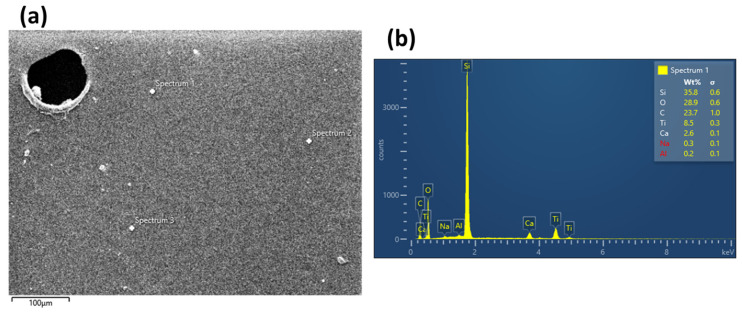
(**a**) SEM micrograph of the as-received silicone membrane surface at 200× magnification; (**b**) Corresponding EDS spectrum obtained from a representative area.

Figure 16 shows the SEM-EDS analysis of the control membrane, exhibiting a generally uniform and homogeneous surface morphology without evidence of deposits or degradation features. The observed void is attributed to an application-induced artifact formed during the multi-layer coating process. The EDS spectrum confirms the expected composition of a PDMS-based system, with Si and O as the primary elements, while Ca and Ti indicate the presence of mineral fillers, consistent with FTIR and TGA observations.

**Figure 17:**
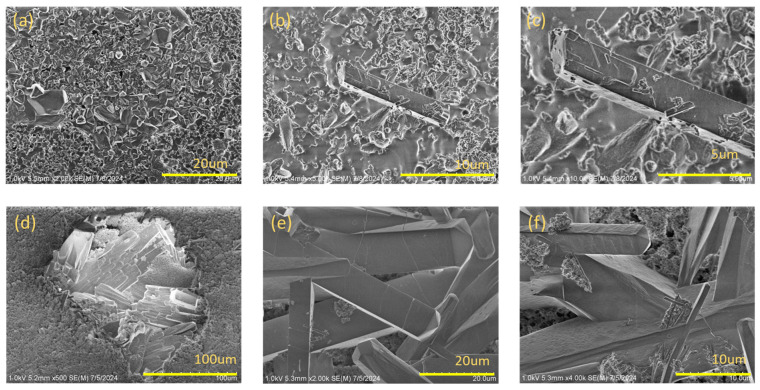
SEM micrographs of silicone membranes after exposure to wet hempcrete at 90 °C for 1 month: (**a**–**c**) non-treated hempcrete (HWNT) and (**d**–**f**) treated hempcrete (HWT).

As shown in Figure 17, SEM observations reveal pronounced surface modification of the silicone membrane following wet exposure to hempcrete, evident even after 1 month of aging. The highest exposure temperature (90 °C) was selected to accentuate morphological changes and capture the most representative degradation features under HWNT-90-1-month and HWT-90-1-month conditions. Images at increasing magnification show the development of surface deposits and morphological changes, including dense crystalline coverage, heterogeneous surface features, and elongated prismatic crystals. Under these conditions, the membrane surface is partially to extensively covered by prismatic and angular crystalline deposits, accompanied by finer granular phases distributed across the surface. At higher magnifications, these features appear as well-defined faceted crystals with clear evidence of aggregation and localized clustering, indicating in situ nucleation and growth on the membrane surface. This behavior suggests that dissolved ionic species released from hempcrete, particularly Ca^2+^ and carbonate species, supersaturate the interfacial suspension and precipitate as mineral phases, such as calcium carbonate, directly onto the membrane.

In addition to mineral deposition, regions exhibiting surface irregularities and localized disruption are observed, suggesting that physical deposition is accompanied by alteration of the underlying polymer matrix. The coexistence of crystalline deposits and disrupted regions suggests a coupled mechanism in which interfacial precipitation and moisture-driven chemical degradation occur simultaneously. Although calcium carbonate deposits are not expected to directly participate in siloxane bond cleavage, their accumulation on the membrane surface may indirectly influence the interfacial environment by altering moisture retention, ion accumulation, and local mass transport pathways. These deposits may therefore promote heterogeneous exposure conditions by retaining moisture and concentrating dissolved ionic species near the membrane surface, while the chemical destabilization of the silicone network is primarily attributed to moisture-enabled hydrolysis and ion-assisted reactions involving dissolved divalent cations. This interplay between mineral deposition and polymer degradation highlights the dynamic nature of the membrane–substrate interface under wet conditions, where both chemical reactions and physicochemical transport processes govern the observed morphology.

**Figure 18:**
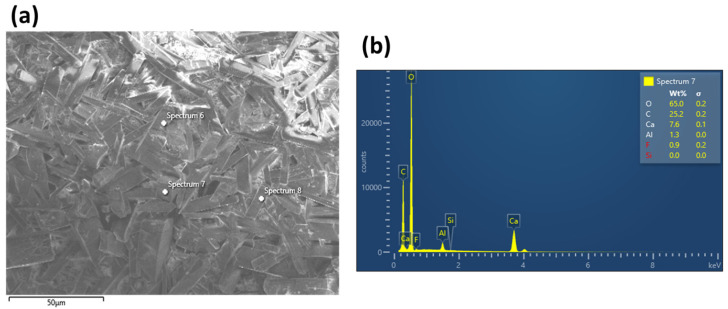
(**a**) SEM micrograph of the elongated prismatic crystalline deposits observed in Figure 17 on silicone membrane surfaces for both HWNT and HWT-90-1-month samples; (**b**) corresponding EDS spectrum obtained from the same crystalline regions.

EDS analysis of the elongated prismatic crystalline features (Figure 18), which were seen in both non-treated and treated samples, shows that these deposits are primarily composed of Ca, O, and C, consistent with calcium-based mineral phases, most likely calcium carbonate. The negligible contribution of Si in these regions confirms that the deposits are not part of the original silicone matrix but are externally formed phases. This interpretation is strongly supported by FTIR results, which show carbonate-related absorption bands, and by TGA data indicating an increase in inorganic residue following exposure. The formation of these deposits is attributed to the precipitation of dissolved calcium species from the hempcrete leachate, driven by supersaturation and carbonation reactions in a moisture-rich environment.

The combined SEM–EDS observations further support the degradation mechanisms inferred from FTIR and DSC analyses. The loss of surface uniformity and the presence of disrupted regions are consistent with chemical degradation of the siloxane network, as evidenced by the attenuation of PDMS-related FTIR bands and the disappearance of the PDMS decomposition peak in TGA. Overall, these results demonstrate that wet exposure induces a coupled degradation process involving both mineral deposition and polymer breakdown, governed by moisture-driven ion transport and chemically reactive leachate conditions at the membrane interface.

## 4. Conclusions

This study evaluated the behavior of silicone membranes in contact with hempcrete under controlled dry and wet aging conditions, with emphasis on understanding the mechanisms governing degradation.

- Membranes exposed to hempcrete under dry conditions showed no significant changes in FTIR, TGA, DSC, or surface morphology, indicating that temperature alone, even up to 90 °C, does not compromise membrane integrity.
- Significant chemical and structural changes were observed under wet conditions as early as one month, confirming that moisture is the primary trigger for membrane deterioration.
- Despite leachate pH values ranging from ~9 to 11, severe degradation occurred across all conditions, indicating that hydroxyl concentration alone does not govern the process. The results suggest that dissolved species, including Ca^2+^ and Mg^2+^ ions, may contribute to ion-assisted hydrolysis and depolymerization of the siloxane network; however, their specific role cannot be fully isolated from the present experiments.
- Membrane deterioration results from the combined effects of chemical degradation (siloxane bond cleavage) and physical constraints imposed by mineral deposition, which restrict polymer chain mobility and suppress thermal transitions observed in DSC.
- Treated hempcrete reduces the aggressiveness of the leachate; however, degradation still occurs under prolonged wet exposure, indicating that treatment alone is insufficient to ensure long-term durability.

Overall, the results demonstrate that the durability of silicone membranes in contact with hempcrete is governed by coupled moisture–chemical interactions rather than thermal effects alone. These findings emphasize the importance of considering leachate composition and moisture exposure in the design and application of membranes in bio-based construction systems. From a practical perspective, prolonged moisture accumulation at the membrane–hempcrete interface should be minimized through appropriate drainage, drying potential, and protection against bulk water ingress. Where sustained wetting or condensation is expected, membrane compatibility should be verified under representative moisture and leachate conditions, and additional measures such as separation layers or alternative membrane systems may be considered.

## Figures and Tables

**Table 1:** Specifications of AllGuard silicone elastomeric coating [20].

Property	Value
Tensile strength (ASTM D412)	1 MPa
Elongation (ASTM D412)	600%
Fungus resistance (ASTM D3274)	No growth
Permeance (ASTM D1653)	2480 m^2^·Pa·s
**Component**	**Content (%)**
Octamethyl cyclotetrasiloxane (D4)	0.032–0.16
Polyether-based additive *	1.1–1.8
Limestone	8.0–11.0

* Simplified name used for readability based on the manufacturer’s description.

**Table 2:** Summary of sample nomenclature and aging conditions.

Exposure Condition	Hempcrete Type	Sample Code	Aging Temperatures	Aging Durations
Control	No hempcrete contact	Control	—	—
Dry	Non-treated shives	HDNT	RT, 50, 70, 90 °C	3, 6, 9 months
Dry	Treated shives	HDT	RT, 50, 70, 90 °C	3, 6, 9 months
Wet	Non-treated shives	HWNT	50, 70, 90 °C	3, 6, 9 months
Wet	Treated shives	HWT	50, 70, 90 °C	3, 6, 9 months

Wet-aged RT samples were excluded from long-term characterization due to visible mold growth on hempcrete after approximately 14 days.

**Table 3:** Summary of characteristic FTIR absorption bands for silicone membranes exposed to hempcrete under wet conditions at different temperatures and exposure durations. “Y”, “W”, and “N” indicate the presence, weak/broad signal, and absence of peaks, respectively.

Sample	Aging Temp (°C)	Aging Time (Month)	FTIR Absorption Bands (cm^−1^)						
			3450	2960	1640	1440	1260	1100–1000	800
**Control**	—	0	N	Y	N	Y	Y	Y	Y
**HWNT**	50	3	W	Y	W	Y	Y	Y	Y
**HWNT**	50	6	Y	W	Y	Y	Y	Y	W
**HWNT**	50	9	Y	Y	W	Y	Y	Y	Y
**HWNT**	70	3	Y	W	Y	Y	Y	W	W
**HWNT**	70	6	Y	W	Y	Y	Y	Y	Y
**HWNT**	70	9	Y	N	Y	N	Y	N	W
**HWNT**	90	3	Y	Y	Y	Y	Y	Y	Y
**HWNT**	90	6	Y	N	Y	Y	N	W	N
**HWNT**	90	9	Y	N	Y	Y	Y	W	N
**HWT**	50	3	Y	Y	W	Y	Y	Y	Y
**HWT**	50	6	Y	Y	W	Y	Y	Y	Y
**HWT**	50	9	Y	Y	Y	Y	Y	Y	Y
**HWT**	70	3	Y	Y	Y	Y	Y	Y	Y
**HWT**	70	6	Y	Y	Y	Y	Y	Y	Y
**HWT**	70	9	Y	Y	Y	N	Y	Y	Y
**HWT**	90	3	W	Y	W	Y	Y	Y	Y
**HWT**	90	6	Y	N	Y	W	N	W	N
**HWT**	90	9	Y	N	Y	Y	N	W	N

**Table 4:** Identified FTIR peaks in the aged membranes.

Peak (cm^−1^)	Assignment
**3450**	O–H stretching (absorbed moisture)
**2960**	C–H stretching (CH_3_ groups in PDMS)
**1640**	H–O–H bending (absorbed moisture)
**1440**	CO_3_^2−^ asymmetric stretching (CaCO_3_ filler/deposition)
**1260**	Si–CH_3_ bending (PDMS backbone)
**1100–1000**	Si–O–Si stretching (siloxane network)
**790**	Si–CH_3_ rocking (PDMS structure)

## Data Availability

The original contributions presented in this study are included in the article/Supplementary Material. Additional raw data supporting the findings of this study have been provided to the publisher and are available from the corresponding author upon reasonable request.

## Supplementary Materials

The following supporting information can be downloaded at: https://www.mdpi.com/article/10.3390/polym18111311/s1, Figure S1. pH changes of suspensions exposed to HWNT samples over its 9-month aging at various temperatures of 50, 70, and 90 °C; Figure S2. pH changes of suspensions exposed to HWT samples over its 9-month aging at various temperatures of 50, 70, and 90 °C.
