# Supplementary material for: Durability of Silicone-Based Waterproofing Membranes in Hempcrete Systems Under Environmental Exposure: Role of Leachate Chemistry and Fiber Treatment
Source: Polymers (Basel). 2026 May 26;18(11):1311. doi: 10.3390/polym18111311 (PMC13258831; doi:10.3390/polym18111311)
Supplement: Supplementary file 1 [file polymers-18-01311-s001.zip › polymers-4301842-supplementary.pdf]

Supplementary Materials

Durability of Silicone-Based Waterproofing Membranes in Hempcrete Systems Under Environmental Exposure: Role of Leachate Chemistry and Fiber Treatment

Elnaz Esmizadeh [1,*], Amir Sabziparvar [1,2], Marzieh Riahinezhad [1], Peter Collins [1], Esrat Jahan [1], Itzel Lopez-Carreon [1] and Donato Taleponga [3]

[1] Building Durability and Resiliency, Construction Research Center, National Research Council Canada,
1200 Montreal Rd, Ottawa, ON K1A 0R6, Canada;
[2] Department of Civil Engineering, University of Ottawa, 161 Louis Pasteur, Ottawa, ON K1N 9K5, Canada
[3] Center of Innovative Technology and Ecodesign (CITÉ), University of Sherbrooke, Sherbrooke, QC J1K 2R1, Canada;
* Correspondence: elnaz.esmizadeh@nrc-cnrc.gc.ca

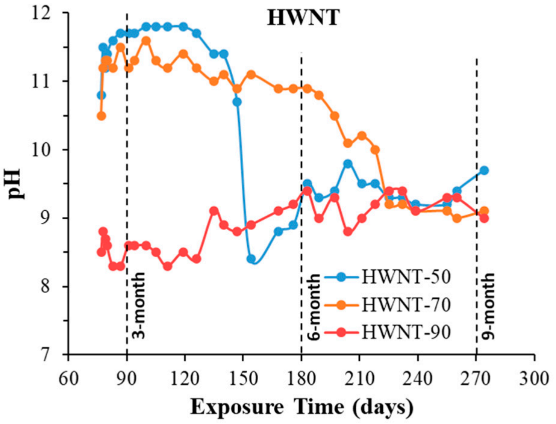

Figure S1. pH changes of suspensions exposed to HWNT samples over its 9-month aging at various temperatures of 50, 70, and 90 °C.

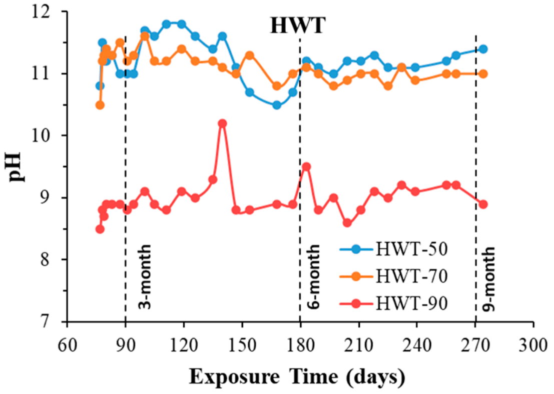

Figure S2. pH changes of suspensions exposed to HWT samples over its 9-month aging at various temperatures of 50, 70, and 90 °C.
